# Physiologically Based Pharmacokinetic Simulation of Tofacitinib in Humans Using Extrapolation from Single-Species Renal Failure Model

**DOI:** 10.3390/pharmaceutics17070914

**Published:** 2025-07-15

**Authors:** Sung Hun Bae, So Yeon Park, Hyeon Gyeom Choi, So Hee Kim

**Affiliations:** 1 College of Pharmacy and Research Institute of Pharmaceutical Science and Technology, Ajou University, Suwon 16499, Republic of Korea; baezzam@ajou.ac.kr (S.H.B.); ggb1212@ajou.ac.kr (H.G.C.); 2 School of Pharmacy and Pharmaceutical Sciences, SUNY-Binghamton University, 96 Corliss Ave, Johnson City, NY 13790, USA; 3 Department of Biohealth Regulatory Science, Graduate School of Ajou University, Suwon 16499, Republic of Korea; qkrth0413@ajou.ac.kr

**Keywords:** tofacitinib, single-species extrapolation, renal failure, PBPK simulation

## Abstract

**Background/Objectives:** Tofacitinib is a Janus kinase 1 and 3 inhibitor that was developed to treat rheumatoid arthritis. Accordingly, this study aimed to predict plasma tofacitinib concentrations and pharmacokinetic parameters in patients with renal failure through physiologically based pharmacokinetic (PBPK) simulations. **Methods**: PK-Sim and Simcyp simulators were used, as well as conventional Dedrick plot analysis, employing a single animal extrapolation method. The predictions were compared with previously published data. **Results:** PBPK simulations of tofacitinib in patients with renal failure closely matched the observed plasma concentration profiles and pharmacokinetic results, including the area under the plasma concentration–time curve (AUC), maximum plasma concentration (*C*_max_), and time to reach *C*_max_ (*T*_max_). The ratios of the simulated to observed plasma concentrations and pharmacokinetic parameters for tofacitinib were within a 0.5–2.0-fold error range. Although the results from the Dedrick plot were reasonably good, they were less accurate than those of the PBPK simulations. This was because the Dedrick plot relied solely on preclinical plasma concentration data without incorporating drug physicochemical properties, in vitro data, or physiological and pathophysiological variables. **Conclusions**: The findings suggest that PBPK simulations using single-species extrapolation effectively provide preliminary estimates of plasma tofacitinib concentration profiles and pharmacokinetic parameters in humans under specific conditions, including renal failure. Furthermore, the results provide a foundation for adjusting tofacitinib dosage and dosing schedules to maintain effective plasma concentrations by considering the pathophysiological characteristics of patients according to their specific diseases.

## 1. Introduction

Physiologically based pharmacokinetic (PBPK) modeling is a computational approach used to predict the absorption, distribution, metabolism, and excretion (ADME) of drugs in humans and animals. This approach integrates physiological parameters, such as organ volumes and blood flow rates, with drug-specific properties, such as solubility, permeability, and metabolic rates, to simulate drug behavior [1]. PBPK models enable mechanistic predictions of drug concentration–time profiles across tissues and organs, thereby facilitating dose selection, risk assessment, and extrapolation from preclinical to clinical settings. The US Food and Drug Administration and European Medicines Agency [2] recognize PBPK simulations as a standard tool to support regulatory evaluations, dosage determinations, and drug applications for specific populations [3,4]. This approach aids in optimizing drug dosing in specific disease conditions and mitigating the risk of drug–drug interactions [1].

**Figure 1:**
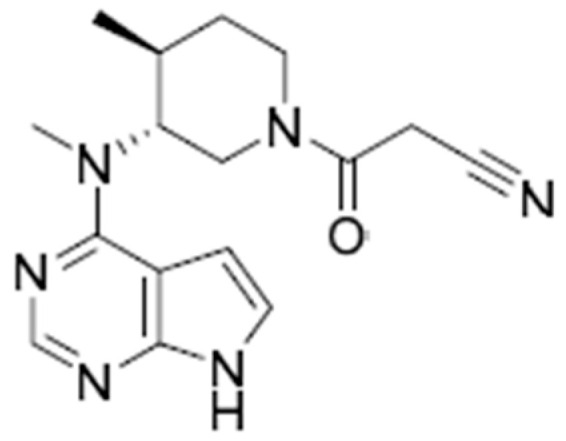
Chemical structure of tofacitinib.

Tofacitinib (Figure 1) inhibits Janus kinases (JAK) 1 and 3, thereby preventing the phosphorylation of signal transducer and activator of transcription (STAT) proteins, which leads to reduced immune cell activation and suppression of pro-inflammatory cytokine signaling pathways [5,6]. It was developed as an oral drug for patients with moderate to severe rheumatoid arthritis who have had an inadequate response to methotrexate [7]. The pharmacokinetic parameters of tofacitinib indicate a half-life of 3.2 h, an absolute oral bioavailability (F) of 74%, and a volume of distribution of 87 L following oral administration of 10 mg in healthy volunteers [8,9,10]. Approximately 70% of tofacitinib undergoes hepatic metabolism via cytochrome P450 (CYP) 3A4 and CYP2C19, whereas the remaining portion is excreted renally as an unmetabolized form [8]. In a preclinical study, male Sprague-Dawley rats were administered 20 mg/kg tofacitinib, and the resulting F value of tofacitinib was 29.1%, with approximately 10.0% of the dose excreted unchanged in the urine [11]. Additionally, approximately 42% of tofacitinib administered via the intraportal vein is metabolized in the liver, whereas approximately 46.1% of the intraduodenally administered drugs are metabolized in the intestine [11]. These findings suggest that the pharmacokinetics of tofacitinib are significantly altered in severe systemic disease conditions.

Kidneys play a critical role in drug elimination [12]. According to the 2017 US Renal Data System, chronic kidney disease has a prevalence of 14.8% among American adults, thus highlighting its magnitude as a common public health concern [13]. Patients with rheumatoid arthritis require long-term therapy; however, nephrotoxic drugs, such as non-steroidal anti-inflammatory drugs and methotrexate, impair renal function. Thus, predicting plasma drug concentrations and adjusting dosage regimens based on the renal function of patients are essential for safe and effective treatment. However, modifying dosage regimens in patients with kidney disease for clinical trials is time-consuming and expensive. As an alternative approach, animal models of kidney disease from a single species can be utilized for human extrapolation and PBPK simulations to predict the plasma concentrations and pharmacokinetics of drugs in patients with renal impairment.

Therefore, this study aimed to predict the plasma concentration and pharmacokinetic parameters of tofacitinib in patients with renal failure using the PK-Sim and Simcyp simulators and a single-species scale-up method for human extrapolation. Additionally, the PBPK simulations were validated by comparing the predicted results with previously published clinical data. Furthermore, a Dedrick plot was used to predict the plasma concentration–time profiles and pharmacokinetics of tofacitinib in patients with renal failure.

## 2. Materials and Methods

### 2.1. Animal Data

The plasma concentration–time profiles and pharmacokinetic parameters of tofacitinib (Table 1) following oral administration (20 mg/kg) in control rats and those with moderate or severe renal failure were obtained from previously published data [14].

### 2.2. Dedrick Plot

The Dedrick plot, which is used to predict the plasma concentration–time profile of drugs in humans, was evaluated in this study. The transformed plasma concentrations of tofacitinib and the corresponding time points were as follows [15]:

$$Transformed\;plasma\;concentration=\frac{Plasma\;concentration\left(\frac{ng}{mL}\right)}{\frac{Dose}{BW}\left(\frac{mg}{kg}\right)}$$

$$Transformed\;time=\frac{Time\left(min\right)}{{BW}^{0.25}}$$

Pharmacokinetic parameters were calculated using the standard non-compartmental analysis method [16] with WinNonlin software version 8.5.2.4 (Certara, NJ, USA). The predicted area under the plasma concentration–time curve (AUC) of tofacitinib in the Dedrick plot was determined using the trapezoidal extrapolation method [17]. The maximum plasma concentration (*C*_max_) and time to reach *C*_max_ (*T*_max_) were obtained directly from the plasma concentration–time profiles.

### 2.3. Extrapolation from Rats to Humans Using Single-Species Method

The total body clearance (CL) in rats (CL_Rat_) was determined from previously published data [18]. CL in humans (CL_Human_) was calculated using single-species extrapolation, following the equation proposed by Tang et al. (2007) [19].

CL_Human_ (mL/kg) = 0.152 × CL_Rat_ (mL/kg)

The coefficient 0.152 was empirically optimized to minimize prediction errors when extrapolating human clearance from rat data.

Renal clearance (CL_R_) in humans (CL_R,Human_) was estimated from the rat CL_R_ (CL_R,Rat_) using the equation described by Paine et al. (2011) [20]:

$${CL}_{R,Human}={CL}_{R,Rat}\times \frac{f_{u,Human}}{f_{u,Rat}}\times \frac{{RBF}_{Human}}{{RBF}_{Rat}}$$

where *f*_u_ and RBF represent the unbound fractions of tofacitinib in the plasma and renal blood flow, respectively. The focus on CL_R_ is due to the significant proportion (approximately 30%) of tofacitinib eliminated via the kidneys [8], making it highly sensitive to changes under renal impairment.

Based on the PK-Sim population data, the RBF values for healthy subjects and patients with moderate and severe renal failure were 18.0, 5.24, and 3.83 mL/min/kg, respectively [21,22]. The corresponding values for rats were 33.3, 28.0, and 18.0 mL/min/kg, respectively [23,24,25,26]. The *f*_u_ value of tofacitinib is 61.0% in humans [27] and 79.3% in rats [28].

Hepatic clearance (CL_H_) was calculated by subtracting CL_R_ from CL. The calculated CL_H_ and CL_R_ values were used as input parameters for the metabolism and excretion of tofacitinib, respectively, and unit conversions were applied according to each software setting.

### 2.4. PBPK Model Development for Tofacitinib

The PBPK simulation model for tofacitinib was developed using PK-Sim (version 11; Bayer Technology Services, Leverkusen, Germany) and Simcyp (version 19; Release 1; Certara, Sheffield, UK) simulators. The physicochemical properties of tofacitinib, including molecular weight, log*p*, pK_a_, and *f*_u_, were obtained from previously published data (Table 2) [2,29,30]. The ADME characteristics of tofacitinib were also obtained from previously reported data (Table 3). A summary of the common input values for the PBPK simulation of tofacitinib is provided in Table 2 and Table 3. The PBPK simulation was verified by comparison with the observed plasma concentration–time profiles in healthy subjects and patients with renal failure, as well as pharmacokinetic parameters, such as AUC, *C*_max_, and *T*_max_ [31]. The plasma concentrations of tofacitinib in patients [31] were extracted by digitizing the reported plasma concentration data using the Engauge Digitizer (version 12.1, https://engauge-digitizer.software.informer.com, accessed on 4 March 2024).

### 2.5. PBPK Model Structure for Healthy Subjects and Patients with Renal Failure

PBPK simulations of the healthy and renal failure models were conducted using PK-Sim and Simcyp following oral administration of 10 mg tofacitinib. The simulations were performed using virtual populations in both PK-Sim and Simcyp.

In PK-Sim, the normal healthy population was defined as Caucasian Americans with no comorbidities or chronic diseases. Based on the estimated glomerular filtration rate (eGRF), the moderate (27.13 < eGFR < 54.25 mL/min/1.73 m^2^) and severe (12.97 ≤ eGFR ≤ 25.94 mL/min/1.73 m^2^) renal failure populations were generated [33] using the embedded virtual population files in the software by selecting the kidney disease status. In Simcyp, the populations were categorized as healthy, moderate (30 < glomerular filtration rate [GFR] < 60 mL/min), or severe (15 < GFR < 30 mL/min) renal failure according to the embedded virtual population files in the software. Considering that eGFR (mL/min/1.73 m^2^) in PK-Sim was calculated based on GFR (mL/min) in Simcyp, the same values were applied to ensure consistency in renal disease conditions across both simulators.

Demographic data for the PBPK simulations were obtained from previously published clinical results [31]. The demographic characteristics, including age (years), height (cm), weight (kg), and body mass index (kg/m^2^) of healthy subjects and patients with renal failure, are presented in Table 4. PBPK simulations were performed with six individuals in PK-Sim, and one trial with six subjects in Simcyp. The male-to-female ratio was set at 50% for both simulators.

**Table 3:** The values of absorption, distribution, metabolism and excretion of tofacitinib in normal subjects and in patients with moderate or severe renal impairment for physiologically based pharmacokinetic (PBPK) simulation using PK-Sim and Simcyp.

	PK-Sim		Simcyp	
	Value	Reference	Value	Reference
**Absorption**				
Intestinal permeability (cm/min)	6.3 × 10^−6^	Predicted based on MDCK cell [34]		
*P*_eff,man_ (cm/s)			22.1 × 10^−6^	Predicted based on Caco-2 cell [34]
**Distribution**				
Partition coefficients	Rodgers and Rowland			
*V*_ss_ (L/kg)	[35]		Predicted	
**Metabolism (CL)**	Type: Plasma CL		Type: In vivo CL	
Normal	5.93	Calculated using single species method [36] (mL/min/kg)	26.9	Calculated using single-species method [37] (L/h)
Moderate renal failure	3.69		17.3	
Severe renal failure	2.23		11.7	
**Excretion (CL_R_)**				
Normal	1.95	Calculated using single-species method (mL/min/kg)	8.86	Calculated using single-species method for healthy volunteer (L/h)
Moderate renal failure	0.202			
Severe renal failure	0.0164			

*P*_eff,man_, effective intestinal permeability in man; *V*_ss_, volume of distribution at steady state; CL, clearance; CL_R_, renal clearance.

### 2.6. Statistical Analysis

All simulated results are presented as mean ± standard deviation, except for T_max_, which is expressed as the median (range). Comparisons between observed and simulated pharmacokinetic parameters were performed using an unpaired Student’s *t*-test, with *p* < 0.05 considered statistically significant.

The ratio (R) between observed and simulated plasma concentrations and pharmacokinetic parameters of tofacitinib in PK-Sim and Simcyp was calculated using the following equation [38]:

$$R\;ratio=\frac{Simulated\;pharmacokinetic\;parameter}{Observed\;pharmacokinetic\;parameter}$$

An R value between 0.5 and 2.0 was considered reasonably reliable and acceptable for the simulated human plasma concentration and pharmacokinetic parameters [39].

To quantitatively compare the plasma concentration–time profiles between observed and simulated values, the difference factor (*f*_1_) method was used [40]. The *f*_1_ value represents the relative error in plasma concentration at each time point, with lower *f*_1_ values indicating higher similarity. This was calculated using the following equation [41]:

$$f_{1}=\frac{\sum_{i=1}^{n}\left|R_{i}-T_{i}\right|}{\sum_{i=1}^{n}R_{i}}\times 100$$

where *n* is the number of time points in the plasma concentration–time curve, and *R*_i_ and *T*_i_ represent the observed and predicted plasma concentrations of tofacitinib at each time point, respectively.

## 3. Results

### 3.1. Human Extrapolation Using Dedrick Plot

**Figure 2:**
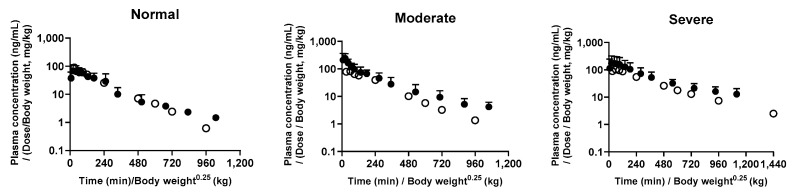
Simple Dedrick plots of tofacitinib plasma concentration–time curve in rats after oral administration of tofacitinib. The closed circles represent the values for humans predicted from the rat data and the open circle is the observed values in humans after oral administration of tofacitinib (10 mg). The x-axis is transformed as time/body weight^0.25^ and the y-axis is transformed as plasma concentration (dose/body weight).

Figure 2 shows the observed [31] and predicted plasma concentration–time profiles of tofacitinib. The relevant pharmacokinetic parameters of tofacitinib are listed in Table 5. The predicted *C*_max_ in healthy subjects decreased by 7.32%, and the corresponding AUC decreased by 10.4% compared with the observed values in normal healthy subjects. The predicted and observed values were comparable. The predicted *C*_max_ in patients with moderate renal failure significantly increased by 144%, and the predicted AUC was 35.1% greater than that observed in these patients. For patients with severe renal failure, the predicted *C*_max_ and AUC values increased by 89.2% and 58.5%, respectively, compared with the observed values. However, these differences were not statistically significant, which implies that the predictions generated by the Dedrick plot analysis are comparable to actual human data, supporting the likelihood that the model can be applied effectively in clinical settings.

### 3.2. PBPK Model Development Using PK-SIM and Simcyp

**Figure 3:**
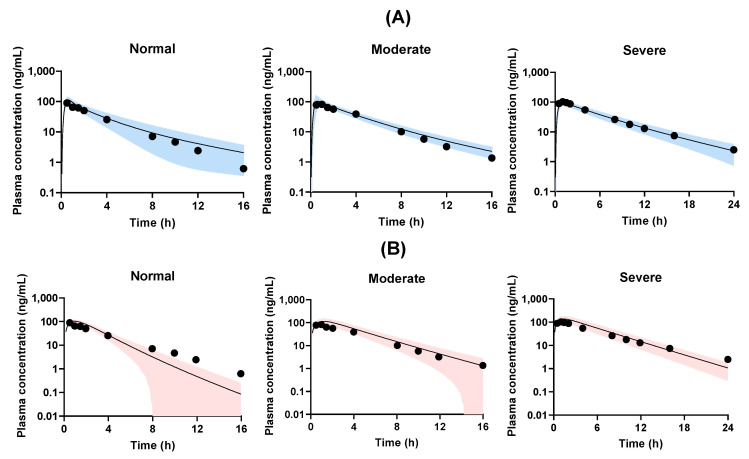
Observed (closed circle) and predicted plasma concentration–time profile of tofacitinib in normal healthy subjects and patients with moderate and severe renal failure using PK-Sim (**A**) and Simcyp (**B**) after oral administration of tofacitinib at a dose of 10 mg. The solid line and the shaded area represent the predicted arithmetric mean and standard deviation, respectively, for the virtual population.

A PBPK simulation of tofacitinib was verified in a published clinical pharmacokinetic study of patients with renal failure [31]. Figure 3 presents the observed and predicted plasma concentration–time profiles of tofacitinib generated using PK-Sim and Simcyp following oral administration of a 10-mg dose. The observed and predicted pharmacokinetic parameters of tofacitinib are listed in Table 5.

The predicted *C*_max_ values increased by 24.2% and 10.4% for PK-Sim and Simcyp, respectively, compared with the observed values in healthy subjects. The corresponding AUC values were 29.5% and 10.4% higher, respectively. In the moderate renal failure model, the predicted *C*_max_ values increased by 14.4% and 10.6% for PK-Sim and Simcyp, respectively, compared with the observed clinical data. The predicted AUC in PK-Sim was comparable to that of the observed data, whereas that in Simcyp was 29.3% higher than the observed clinical data for moderate renal failure. In the severe renal failure model, the predicted *C*_max_ decreased by 11.2% in PK-Sim but increased by 25.3% in Simcyp compared with the observed clinical data. The predicted AUC in PK-Sim was comparable to that of the observed data, whereas that in Simcyp was 34.3% higher. Overall, the predicted AUC values tended to be higher than those of the observed clinical data, except for the PK-Sim simulations for moderate and severe renal failure.

Interestingly, in PK-Sim, the predicted *C*_max_ in patients with severe renal failure decreased compared with that in normal healthy subjects, an opposite trend. Similarly, Li et al. (2022) [42] reported that the *C*_max_ of schaftoside, a 3-hydroxyflavone from Desmodium styracifolium that prevents gallstones and kidney stones, decreased in renally impaired patients; however, PBPK simulation using Simcyp revealed that the AUC increased as chronic kidney disease stages progressed. The predicted AUCs were generally higher than those of the observed clinical data, except in the PK-Sim simulation of moderate and severe renal failure. Similar results were reported by Edginton and Willmann (2008) [43] for PBPK simulations of lidocaine in patients with liver cirrhosis.

### 3.3. Predicted Model Validation for Renal Failure Model

**Figure 4:**
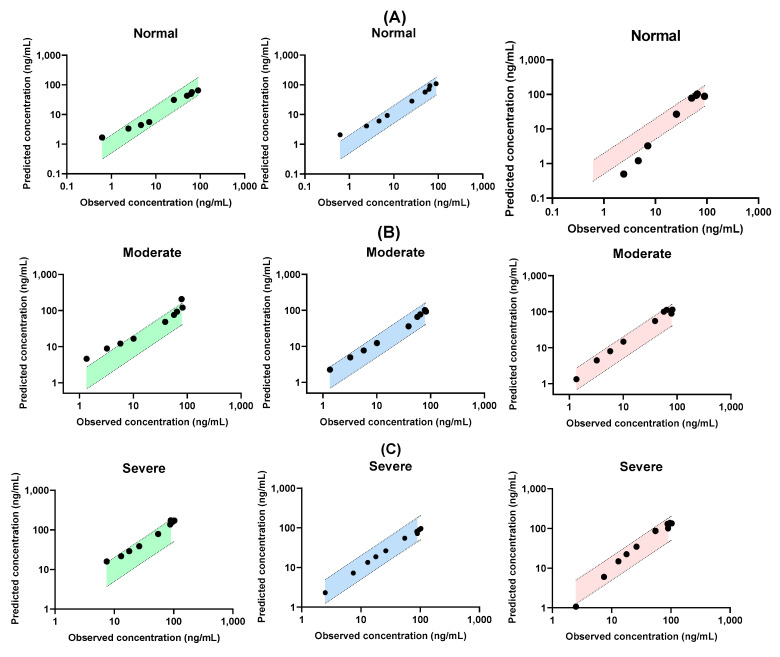
Correlation between observed and predicted plasma concentration of tofacitinib in normal healthy subjects and patients with moderate and severe renal failure using Dedrick plot (**A**), PK-Sim (**B**) and Simcyp (**C**), respectively, after oral administration of tofacitinib at a dose of 10 mg. The closed circle is the predicted value and the dashed line with the shaded area represents a within-two-fold error of the observed data.

**Figure 5:**
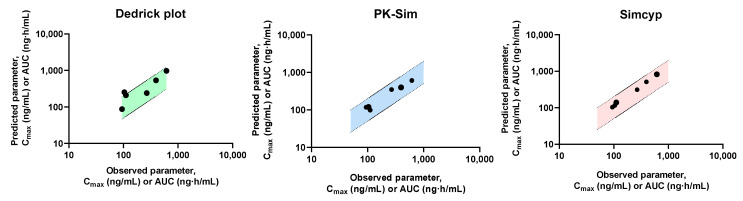
Comparison of observed and predicted pharmacokinetic parameters, AUC and *C*_max_, of tofacitinib after the Dedrick plot, PK-Sim and Simcyp. The closed circle is the predicted value and the dashed line with the shaded area represents a within-two-fold error of the observed data using Dedrick plot, PK-Sim and Simcyp, respectively, after oral administration of tofacitinib at a dose of 10 mg. *C*_max_, the peak plasma concentration; AUC, total area under the plasma concentration–time curve from time zero to last time.

Figure 4 shows the observed and predicted plasma concentrations of tofacitinib at the same time points. Among the nine predicted plasma concentration time points in normal subjects, eight were within a 0.5–2.0-fold error range of the observed data when using the Dedrick plot and PK-Sim, whereas five were within this range when using Simcyp. In the moderate renal failure model, five, nine, and nine of the nine predicted plasma concentration time points were within a 0.5 to 2.0-fold error range of the observed data when using the Dedrick plot, PK-Sim, and Simcyp, respectively. In the severe renal failure model, all predicted plasma concentrations of tofacitinib, except for one time point, were within a 0.5–2.0-fold error range of the observed data when using the Dedrick plot and Simcyp. All predicted plasma concentration time points were within this error range when using PK-Sim. In addition, the predicted pharmacokinetic parameters, including *C*_max_ and AUC, when using the Dedrick plot and both simulators in normal subjects and patients with moderate and severe renal failure, were within a 0.5–2.0-fold error range of the observed data, except for *C*_max_ in the moderate renal failure model when using the Dedrick plot (Figure 5). This suggests that the predictions for both healthy subjects and patients with renal failure using single-species extrapolation are comparable with the observed data and can be considered acceptable from an industrial standpoint [44].

To validate the simulation model for predicting the pharmacokinetic parameters of tofacitinib, *f*_1_ values were calculated (Table 6). The *f*_1_ values in the Dedrick plot were 37.3%, 101%, and 68.5% for normal renal function, moderate renal failure, and severe renal failure, respectively. This indicated that the predictions in the healthy subjects were more similar to the observed data than those in the moderate and severe renal failure models. The *f*_1_ values in the PK-Sim group were 51.9%, 29.3%, and 6.02% for normal, moderate, and severe renal failures, respectively. The corresponding values for Simcyp were 51.5%, 41.1%, and 34.2%, respectively. These results suggest that the predictions based on the Dedrick plot provided reasonable accuracy and were comparable to those obtained with PK-Sim or Simcyp in subjects with normal renal function. However, its predictive performance declined under renal impairment conditions compared to PBPK simulations, with PK-Sim and Simcyp yielding predictions more consistent with the observed data in patients with severe renal failure.

## 4. Discussion

Among the various modeling and simulation approaches, the bottom-up method was used for predictions in this simulation. The bottom-up method is a simulation-based modeling approach that combines the physiological and physicochemical properties of a drug with system-dependent parameters such as organ volume, blood flow rate, enzyme and transporter expression level, and plasma protein binding from humans and preclinical species. These inputs are applied to a modeling platform to construct mechanistic models that describe the processes of absorption, distribution, metabolism, and excretion. Using preclinical and in vitro data, this approach allows prediction of pharmacokinetic behavior in humans without relying on prior clinical information [1,45]. A key advantage of this strategy is its ability to predict human plasma concentrations in the early stages of new drug development under various disease conditions using preclinical data alone without requiring any clinical data [46].

In the PBPK simulation of tofacitinib in patients with renal failure, an oral dose of 10 mg was administered because this dosage is commonly prescribed for patients with rheumatoid arthritis [47]. In rheumatoid arthritis, comorbid conditions affecting other organs, such as the kidneys, intestines, and liver, may be present [48,49]. Therefore, adjusting the dosage and dosing interval according to the patient’s condition is essential.

To predict the plasma concentration and pharmacokinetic parameters of tofacitinib in patients with renal failure based on previously reported pharmacokinetic parameters in rats [18], a single-species extrapolation method was applied [44,50]. Gentamicin and cisplatin induce moderate and severe histological damage, respectively, in rat kidneys [51,52]. Thus, in this study, rats with gentamicin-induced moderate renal failure and cisplatin-induced severe renal failure from a previous study [18,53] were used as models to predict tofacitinib plasma concentrations and pharmacokinetic parameters in humans with moderate and severe renal failure, respectively.

Using the Dedrick plot, the *R* ratios generally ranged from 0.5 to 2.0; however, several deviations from the PBPK simulations were observed in terms of the plasma concentration and pharmacokinetic parameters. This may be because the Dedrick plot is a simple approach that requires only preclinical plasma concentration–time profiles [54], unlike PBPK simulation. Additionally, the single-species extrapolation method cannot be applied to a complex Dedrick plot, which requires data from at least three animal species and provides more accurate predictions of human plasma concentration–time profiles and pharmacokinetic parameters [55]. In contrast, PBPK simulation requires additional input, including the physicochemical properties of the drug, in vitro data, and physiological and pathophysiological variables [56]. The PBPK model incorporates various physiological assumptions, including perfusion-limited tissue distribution, standardized organ volumes and blood flows, and enzymatic activity, all of which are represented through systems of ordinary differential equations [56]. Each assumption is based on known anatomical and physiological characteristics of the human body and is appropriately adjusted for special conditions such as renal impairment. Hence, although the Dedrick plot provided relatively good predictions, the *f*_1_ values were higher than those obtained from the PBPK [57].

Therefore, PBPK simulations have the potential to serve as an alternative to clinical experiments, while contributing to translational research and supporting pharmaceutical regulatory evaluation [58]. Data on the physicochemical properties of tofacitinib were obtained from the literature. The CL_Human_ and CL_R,Human_ values are typically estimated using the conventional allometry method, which requires data from at least three animal species [59]. However, in this study, CL_Human_ was predicted using a single-species approach [19], and CL_R,Human_ was calculated by applying correction factors, including plasma protein binding and RBF rate [20]. This approach allows for the prediction of CL_Human_ and CL_R,Human_ with limited preclinical data from a single [60].

In general, decreased CL indicates slower metabolism and/or reduced renal excretion [16]. CL was estimated as the sum of CL_H_ and CL_R_, assuming that tofacitinib is metabolized exclusively in the liver [61]. Because renal failure not only affects renal excretion parameters, such as CL_R_ and glomerular filtration rate (GFR), but also reduces hepatic metabolism [62], CL_H_ was also considered in this simulation. Reduced mRNA expression levels of CYP [63] and CYP enzyme activity [64] have been observed in patients with renal failure. Hence, these findings suggest that alterations in hepatic enzymes may influence the overall CL of tofacitinib in renal failure models.

The discrepancy between the observed and simulated plasma concentrations of tofacitinib was mainly observed in the later phase of elimination rather than in the absorption phase. For example, in one of the study data points, the predicted plasma concentration of tofacitinib in PK-Sim (28.2 ng/mL) was 10.2% higher than the observed concentration (25.6 ng/mL) at 4 h after oral administration to normal subjects. The discrepancy was even greater at 16 h post-administration when the simulated concentration in PK-Sim (2.08 ng/mL) exceeded the observed value (0.620 ng/mL) by 235%. One possible reason for this discrepancy is that the observed plasma concentration–time profile of tofacitinib was obtained from a previously published study using an extraction program [55]. Thus, this method may have introduced minor errors in reading the graph plots, unlike the pharmacokinetic parameters obtained directly from the literature. Consequently, relatively lower plasma concentrations resulted in higher *f*_1_ values, particularly in the Dedrick plot simulation when compared with the PK-Sim and Simcyp simulations. A similar trend regarding the differences in the late elimination phase has been reported in a renal failure PBPK model for the antidiabetic drug repaglinide [35]. Another possible explanation for the greater discrepancy in late-phase plasma concentrations could be measurement errors in the observed plasma concentration of tofacitinib using analytical equipment. However, the lower limit of quantitation for tofacitinib in human plasma has been reported as 0.5 ng/mL [65], which is sufficient to quantify the lowest observed clinical plasma concentration (0.620 ng/mL in normal subjects). Therefore, the inaccuracy of the analytical machinery used to determine the tofacitinib concentrations was considered negligible in this simulation. Overall, the results demonstrated close agreement between the observed and simulated data, particularly in terms of *C*_max_ and AUC values.

The differences between the PK-Sim and Simcyp results could be attributed to variations in the input values, such as permeability during absorption. In PK-Sim, the MDCK cell permeability value was applied, whereas in Simcyp, Caco-2 cell permeability was used. The available input options, which also include MDCK II and LLC-PK1, depend on the simulator platform. Although the distribution parameter differs between the two simulators, its effects on the results were minimal because both Simcyp and PK-Sim applied the ‘Rodgers and Rowland’ method.

The R ratios for pharmacokinetic parameters were within two-fold in the Dedrick plot (except for *C*_max_ in the moderate renal failure model) and in the PBPK simulations using PK-Sim and Simcyp. However, these results have some limitations. First, Lin and Wong (2017) [66] reported that oral absorption of drugs depends on various drug properties, such as particle size, salt form, and dosage form. In this study, tofacitinib was considered to be an immediate-release formulation that could influence drug bioavailability depending on its dissolution time. Second, the single-species extrapolation method used in this simulation may not be applicable to all drugs. For example, an equation for CL was developed by excluding idarubicin because of clear outlier data [19]. Additionally, the CL_R_ estimation method based on single-species data is limited to drugs that are actively secreted into the urine via organic anion transporters. This suggests that extrapolating the CL values from a single species may not be appropriate for certain drugs [20,67]. Third, the effects of food were not considered in the simulation development. The AUC and C_max_ of alectinib markedly increased under fed conditions, whereas the pharmacokinetic parameters of isoniazid markedly decreased compared to the fasted state [68]. Furthermore, the PBPK simulation results for pazopanib, an anticancer angiogenesis inhibitor, showed opposite trends in the *C*_max_ and AUC ratio (fed/fasted) between the observed and predicted values [68]. Lastly, the contribution of transporter-mediated processes, such as OAT transporters or the mechanisms underlying potential drug–drug interactions, was not explicitly incorporated into the PBPK model due to a lack of quantitative in vitro or clinical data. Therefore, further studies are required to incorporate these additional factors into more comprehensive simulations.

## 5. Conclusions

The pharmacokinetic parameters of tofacitinib, such as AUC and *C*_max_, were comparable between the observed and simulated data in a renal failure model utilizing the Dedrick plot, PK-Sim, and Simcyp. This study suggests that these simulations were accurately predicted using a single-species extrapolation method and serve as a valuable tool for forecasting the pharmacokinetics of drugs in populations with diseases for which clinical trial data are lacking. This further indicates that these methods can effectively predict tofacitinib plasma concentration profiles and pharmacokinetic parameters in humans based on given data conditions using various simulation methods. Furthermore, the study results provide a foundation for adjusting drug dosage and dosing schedules to maintain effective plasma concentrations by considering the pathophysiological characteristics of patients according to their specific diseases.

## Figures and Tables

**Table 1:** Pharmacokinetic parameters of tofacitinib following intravenous administration at a dose of 10 mg/kg in normal rats, and in rats with moderate or severe renal impairment induced by gentamicin or cisplatin, respectively.

	Normal (*n* = 6)	Moderate (*n* = 8)	Severe (*n* = 7)
Body weight (g)	280 ± 19.0	251 ± 21.3	188 ± 10.2
AUC (μg·min/mL)	264 ± 45.4	433 ± 90.0	693 ± 105
CL (mL/min/kg)	39.0 ± 7.97	24.3 ± 6.95	14.7 ± 2.29
CL_R_ (mL/min/kg)	4.75 ± 1.28	1.45 ± 1.54	00679 ± 0.0917

The data were obtained from the study conducted by Bae et al. 2022 [14].

**Table 2:** Basic physicochemical properties of tofacitinib for physiologically based pharmacokinetic (PBPK) simulation using PK-Sim and Simcyp.

Physicochemical Properties	Value	Reference
Molecular weight (g/mol)	312.4	[29]
Log*p* *	1.15	[30]
pK_a_	5.07	[32]
*f*_u,p_	0.61	[2]

log*p*, log of the permeability; pK_a_, negative log of the dissociation constant; B/P ratio, blood-to-plasma partition ratio; *f*_u,p_, unbound fraction of a drug in plasma. * Log*p* value of 1.15 as the free base form of tofacitinib

**Table 4:** Demographic characteristics for normal subjects and patients with moderate or severe renal impairment.

	Normal	Moderate	Severe
Age (years)	37–65	37–63	31–72
Height (cm)	165–193	160–175	155–175
Weight (kg)	65–87	65–116	74–109
BMI (kg/m^2^)	21–29	23–41	27–40

The data were obtained from the study conducted by Krishnaswami et al., 2014 [31].

**Table 5:** Mean values (±standard deviation) of observed (*n* = 6 for each of the normal, moderate, and severe groups) and predicted (*n* = 6, 8, and 6 for normal, moderate, and severe groups, respectively) pharmacokinetic parameters of tofacitinib using the Dedrick plot, PK-Sim, and Simcyp in normal subjects and patients with moderate or severe renal impairment.

	Parameters	Observed	Dedrick Plot	PK-Sim	Simcyp
Normal	*C*_max_ (ng/mL)	94.2 ± 25.3	87.3 ± 30.4	117 ± 25.4	104 ± 18.9
	AUC (ng∙h/mL)	268 ± 71.5	240 ± 26.5	347 ± 141	312 ± 78.8
	*T*_max_ (h)	0.75 (0.50–1.50)	1.39 (0.112–4.08)	0.600 (0.50–0.95)	0.937 (0.866–1.16)
Moderate	*C*_max_ (ng/mL)	104 ± 47.5	254 ± 136 *	119 ± 53.4	115 ± 39.3
	AUC (ng∙h/mL)	396 ± 154	535 ± 269	397 ± 97.9	512 ± 203
	*T*_max_ (h)	0.75 (0.50–2.00)	0.372 (0.121–1.13)	0.800 (0.450–1.15)	1.16 (1.04–1.39)
Severe	*C*_max_ (ng/mL)	111 ± 28.6	210 ± 152	98.6 ± 23.1	139 ± 48.0
	AUC (ng∙h/mL)	615 ± 214	975 ± 551	608 ± 161	826 ± 344
	*T*_max_ (h)	0.75 (0.50–1.50)	0.777 (0.378–3.12)	0.600 (1.08–1.30)	1.33 (1.20–1.44)

AUC, area under the plasma concentration–time curve from time zero to last time; C_max_, the peak plasma concentration; *T*_max_, time to reach *C*_max_. * *p* < 0.05 mean significantly different from observed data.

**Table 6:** The *f*_1_ value (%) of relative error for plasma concentration curves of tofacitinib in normal subjects and patients with moderate or severe renal impairment using the Dedrick plot, PK-Sim and Simcyp.

*f*_1_ Value (%)	Normal	Moderate	Severe
Dedrick plot	37.3	101	68.5
PK-Sim	51.9	29.3	6.02
Simcyp	51.5	41.1	34.2

## Data Availability

All data described in the study can be found in the article, and we do not have any supporting data.
